# Machine Learning-Augmented Propensity Score-Adjusted Multilevel Mixed Effects Panel Analysis of Hands-On Cooking and Nutrition Education versus Traditional Curriculum for Medical Students as Preventive Cardiology: Multisite Cohort Study of 3,248 Trainees over 5 Years

**DOI:** 10.1155/2018/5051289

**Published:** 2018-04-15

**Authors:** Dominique J. Monlezun, Lyn Dart, Anne Vanbeber, Peggy Smith-Barbaro, Vanessa Costilla, Charlotte Samuel, Carol A. Terregino, Emine Ercikan Abali, Beth Dollinger, Nicole Baumgartner, Nicholas Kramer, Alex Seelochan, Sabira Taher, Mark Deutchman, Meredith Evans, Robert B. Ellis, Sonia Oyola, Geeta Maker-Clark, Tomi Dreibelbis, Isadore Budnick, David Tran, Nicole DeValle, Rachel Shepard, Erika Chow, Christine Petrin, Alexander Razavi, Casey McGowan, Austin Grant, Mackenzie Bird, Connor Carry, Glynis McGowan, Colleen McCullough, Casey M. Berman, Kerri Dotson, Tianhua Niu, Leah Sarris, Timothy S. Harlan, on behalf of the CHOP Co-investigators

**Affiliations:** ^1^The Goldring Center for Culinary Medicine, Tulane University School of Medicine, 300 N. Broad St., Suite 102, New Orleans, LA 70119, USA; ^2^Tulane University School of Public Health & Tropical Medicine, New Orleans, LA, USA; ^3^Texas Christian University, Fort Worth, TX, USA; ^4^Texas College of Osteopathic Medicine, Fort Worth, TX, USA; ^5^University of Texas School of Medicine in San Antonio, San Antonio, TX, USA; ^6^Rutgers Robert Wood Johnson Medical School, New Brunswick, NJ, USA; ^7^Lake Erie College of Osteopathic Medicine, Arnot Ogden Medical Center, Erie, PA, USA; ^8^Meharry Medical College, Nashville, TN, USA; ^9^University of Illinois-Chicago College of Medicine, Chicago, IL, USA; ^10^University of Colorado-Denver School of Medicine, Denver, CO, USA; ^11^Western University of Health Sciences College of Osteopathic Medicine of the Pacific-Northwest, Lebanon, OR, USA; ^12^University of Chicago Pritzker School of Medicine, Chicago, IL, USA; ^13^Pennsylvania State University College of Medicine, Hershey, PA, USA

## Abstract

**Background:**

Cardiovascular disease (CVD) annually claims more lives and costs more dollars than any other disease globally amid widening health disparities, despite the known significant reductions in this burden by low cost dietary changes. The world's first medical school-based teaching kitchen therefore launched CHOP-Medical Students as the largest known multisite cohort study of hands-on cooking and nutrition education versus traditional curriculum for medical students.

**Methods:**

This analysis provides a novel integration of artificial intelligence-based machine learning (ML) with causal inference statistics. 43 ML automated algorithms were tested, with the top performer compared to triply robust propensity score-adjusted multilevel mixed effects regression panel analysis of longitudinal data. Inverse-variance weighted fixed effects meta-analysis pooled the individual estimates for competencies.

**Results:**

3,248 unique medical trainees met study criteria from 20 medical schools nationally from August 1, 2012, to June 26, 2017, generating 4,026 completed validated surveys. ML analysis produced similar results to the causal inference statistics based on root mean squared error and accuracy. Hands-on cooking and nutrition education compared to traditional medical school curriculum significantly improved student competencies (OR 2.14, 95% CI 2.00–2.28, *p* < 0.001) and MedDiet adherence (OR 1.40, 95% CI 1.07–1.84, *p* = 0.015), while reducing trainees' soft drink consumption (OR 0.56, 95% CI 0.37–0.85, *p* = 0.007). Overall improved competencies were demonstrated from the initial study site through the scale-up of the intervention to 10 sites nationally (*p* < 0.001).

**Discussion:**

This study provides the first machine learning-augmented causal inference analysis of a multisite cohort showing hands-on cooking and nutrition education for medical trainees improves their competencies counseling patients on nutrition, while improving students' own diets. This study suggests that the public health and medical sectors can unite population health management and precision medicine for a sustainable model of next-generation health systems providing effective, equitable, accessible care beginning with reversing the CVD epidemic.

## 1. Introduction

The global leader among mortality causes is cardiovascular disease (CVD), claiming the lives of over 1 in 3 people annually throughout the world and nearly 1 in 5 dollars of the total United States health expenditures [1, 2]. This epidemic for both medical and public health sectors is only projected to increase, as CVD is expected to affect nearly half the population with at least double the cost by 2035 [3, 4]. Yet nearly 40% of CVD is attributed to modifiable social determinants of health including poor diet, exercise, and smoking which worsen endothelial function and arterial compliance [5]. These trends are further complicated by health disparities including racial and sexual [6]. Aside from the ethical toll, health disparities every year account for over 1 in 10 health expenditure dollars or $1.24 trillion every three years [7, 8].

Amid this backdrop, diet-driven improvements in such nutrition-related CVD risk factors as obesity, diabetes, and hypertension have been shown in a simulated trial with the Archimedes Model to reduce the collective risk of myocardial infarctions and stroke by 46% under real-world conditions [9]. The Mediterranean Diet (MedDiet) in particular has increasingly emerged in multiple systematic reviews and meta-analyses of randomized controlled trials (RCTs) and large cohorts as a clinically effective and low-cost intervention to improve CVD risk factors and also mortality [10–16]. However only 1 in 2 primary care physicians regularly educate their patients on nutrition [17], while over 7 out of 10 American medical school graduates report inadequate training in how to provide nutrition counseling for patients [18].

Not only nutrition, but more broadly medical education has failed to keep pace with some of the most fundamental shifts in medicine and public health to match global patient health needs particularly with CVD—there are no known medical education institutions actively training future physicians in the societal transition from the Information Age to the Age of Artificial Intelligence (AI) [19]. AI is rapidly transforming nearly every aspect of society in clinically relevant ways, including how patients' outcomes are impacted by finances, education, infrastructure, politics, and ecology [20]. AI through machine learning (ML) self-learning algorithms are playing an increasingly pivotal role in precision medicine by accelerating the speed and accuracy of cost-effective health system interventions to improve population health from both the medical and public health sectors [21–23]. A recent demonstration of this potential is the collaboration between Mayo Clinic and UnitedHealth Group to create Optum Labs, allowing an unprecedented clinical and claims dataset of 150 million patients, compliant with the Health Insurance Portability and Accountability Act (HIPAA) [24]. By rapidly integrating -omics, clinical, and claims datasets, AI is advancing the synergistic role of precision medicine and population health to improve patient outcomes through improved diagnostics and treatments by clinicians, resource management by learning health system executives and policymakers, and ultimately more efficient and ethical outcomes generated for patient populations by the medical and public health sectors [19–25].

Driven by these larger trends, the Goldring Center for Culinary Medicine (GCCM) at Tulane University School of Medicine (GCCM) was created in 2012 as the world's first known medical school-based teaching kitchen to reverse the CVD epidemic through sustainable, scalable culinary medicine programs. GCCM increasingly has incorporated ML to increase the speed and accuracy identifying at-risk patients in addition to optimal intervention, study design, and implementation aspects in culturally and socioeconomically diverse communities throughout America. By first training medical students in nutrition education through hands-on cooking classes in a lower-income former food desert-based grocery store complex, GCCM provides free cooking and nutrition classes to community members, taught by those students. The force multiplying effect of this two-part education model was intended to train a more well-equipped generation of physicians, while providing clinically efficacious, financially sustainable, culturally-sensitive cooking and nutrition classes throughout America's health system particularly with the ultimate goal of reversing the CVD epidemic.

To establish the evidence-base foundation for this nutrition curriculum, GCCM through its collaboration network of 45+ medical schools, colleges, and hospitals launched Cooking for Health Optimization with Patients (CHOP). This study serves as the largest and longest-running known multisite cohort trial on hands-on cooking and nutrition education for medical students, in addition to the first known to incorporate causal inference traditional statistics augmented by ML. Previous attempts to test efficacy for similar interventions have been limited by no validated surveys tools [26–29], multisite design [28–32], long-term follow-up [26–28, 30–32], or adequately powered sample sizes [27–33]. CHOP therefore seeks to test causal inference if hands-on cooking and nutrition education compared to standard of training and care for medical trainees (through the cohort study) and patients (through nested Bayesian adaptive randomized trials), respectively, can improve trainee competencies in providing patients nutrition education and patient outcomes. The study reported here is a substudy within CHOP, CHOP-Medical Students, with the objectives of determining if hands-on cooking and nutrition education versus traditional education for medical students can have inferred causality to improve student competencies and attitudes about providing patients with nutrition education along with students' own diet and if such a program could be scalable.

## 2. Methods

### 2.1. Study Design

Due to the logistical and ethical prohibitive challenges, a randomized controlled trial design was not implemented. Rather, CHOP-Medical Students features a prospective multisite cohort study design. Inclusion criteria were any medical student responder to the validated survey [34] sent electronically to the first 20 GCCM collaborating medical schools licensing the GCCM curriculum from August 1, 2012, to June 26, 2017, stretching from Oregon to Pennsylvania and Chicago to New Orleans. Exclusion criteria were any responder who completed more than one survey per survey cycle within each semester or failed to report number of GCCM classes received if any.

### 2.2. Control and Treatment

Control was the standard medical school curriculum, which was assumed to be adequately homogenous to allow comparison due to the common accreditation standards outlined by the United States Department of Education-recognized Liaison Committee on Medical Education, jointly sponsored by the American Medical Association (AMA) and the Association of American Medical Colleges (AAMC) [35]. Further, there is no evidence-based nutrition education widely provided within school curricula nationally, with the wide majority not meeting even the minimum nutrition hours outlined by the National Academy of Sciences (NAS) of 25 [36]. Treatment was the GCCM nutrition education curriculum, designed and implemented as a supplement to school curricula, annually updated based on the GCCM CHOP collaborating schools and latest literature [34]. The GCCM curriculum utilizes the active learning approach of hands-on cooking and nutrition education in medical school-based teaching kitchens, in contrast to solely utilizing the lecture or small group-based format of nutrition teaching in traditional medical school curriculum. This active approach, indicated in a recent meta-analysis to be superior to traditional clinical education [37], translates the latest evidence-based medical education model of simulation-based medical education with deliberate practice (SBME-DP) into training for patient nutrition counseling. To exceed the NAS minimum, the GCCM elective curriculum consists of 28 hours of instruction over 8 classes, each divided up by 0.5-hour preclass lecture videos, 1.5 hours of hands-on cooking, and 0.75-hour postclass problem-based learning (PBL) sessions as the trainees eat their prepared meals. These sessions serve to foster clinical application of the lecture material, constructed based on national board exam questions.

### 2.3. Study Endpoints

**Figure 1:**
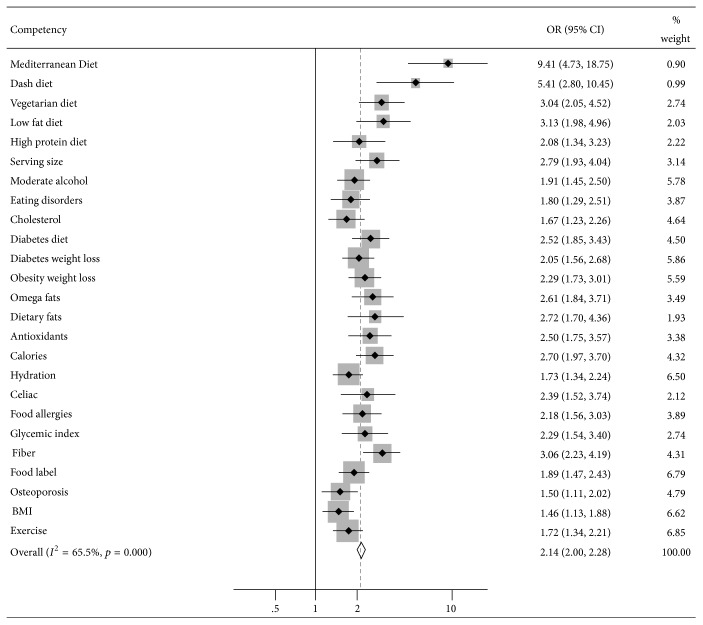
CHOP-Medical Students (*N* = 3,248): inverse-variance weighted fixed effects meta-analysis of propensity score-adjusted multilevel mixed effects regression panel analysis of hands-on cooking and nutrition education versus control for counseling patients on key nutrition topics.

The primary endpoint was 20% increased odds of medical trainees achieving competency mastery educating patients on 25 nutrition topics selected based on the above literature and their clinical significance to improve patient outcomes (Figure 1, Table 1). The secondary endpoint was 10% increased odds of medical trainees' high and medium versus low adherence to the MedDiet. These endpoints were selected based on the existing literature on medical student nutrition education [26–33] including the first phase of this trial [34] and the clinically significant threshold to pragmatically justify an educational intervention for trainees with the ultimate purpose of producing superior patient outcomes.

### 2.4. Data Source

The primary data source was the odd-number, Likert scale-based survey described above. Ethical standards required no coercion for survey completion including no threat of negative impact on student grades, and so it had to remain voluntary. Subsequently there was notable incomplete follow-up at the individual level and incomplete time to track all individuals joining the study rapidly from new collaborating sites from their first through fourth years of schooling. The MedDiet score utilized in the survey was adapted from the 9-point scale described in the seminal* New England Journal of Medicine* article by Trichopoulou et al. [14, 15].

### 2.5. Power Analysis

The required calculated sample size to achieve 80% power and detect an odds ratio (OR) of at least 1.50 with a two-sided test set at 5% was 263 cases.

### 2.6. Machine Learning and Statistical Analysis

The first phase of the analysis was conducted with ML within a supervised learning framework to test 43 algorithms with 10-fold cross-validation, selected based upon the data type. Algorithm performance was assessed favorably based on higher accuracy, lower root relative squared error (RRSE) with model acceptability set at 100% (for comparison among ML algorithms), and lower root mean squared error (RMSE, for comparison to traditional statistical models) [38]. Performance could be improved further if the relevant ML algorithms would be additionally permitted to select which variables should be included in the models. But to improve comparison to traditional statistical results, all the variables based on the above literature to be included in the statistical regression models were included first in the ML algorithms. The following algorithms by type were tested: Bayesian (Bayes Net, Naive Bayes, Naive Bayes Multinomial Text, and Naive Bayes Updateable), Functions (Logistic, Multilayer perceptron, SGD, SGD Text, Simple Logistic, SMO, and Voted Perceptron), Lazy (IBK, KStar, and LWL), Meta (AdaBoostM1, Attribute Selected Classifier, Bagging, Classification via Regression, CV Parameter Selection, Iterative Classifier Optimizer, Logit Boost, Multiclass Classifier, Multiclass Classifier Updateable, Multi-Scheme, Random Committee, Randomizable Filtered Classifier, Random Sub-Space, Stacking, Vote, and Weighted Instances Handler Wrapper), Miscellaneous (Input Mapped Classifier), Rules (Decision Table, JRip, OneR, Part, and ZeroR), and Trees (Decision Stump, Hoeffding Tree, J48, LMT, Random Forest, Random Tree, and REP Tree).

The second phase of analysis was conducted with traditional statistics using a novel integration of three statistical methods. Panel analysis of longitudinal data with triply robust propensity score (PS) adjusted multilevel mixed effects multivariable regression was conducted for causal inference. There is extensive, well-accepted statistical literature that causation can be inferred in observational trials without randomization through certain rigorous statistical methods, with fixed effects and propensity score being two of the most popular and well validated particularly with panel data [39–43].

Doubly robust estimation features a dual strategy of outcome regression that also accounts for the likelihood of receiving an exposure or treatment, such as with a PS [39, 44–46]. Simply using multivariable regression or PS analysis can lead to biased treatment estimates if either model is incorrectly specified. But with doubly robust estimation using both concurrently, only one model must be correctly specified to produce unbiased estimates. Of all the forms of PS analysis (i.e., matching, stratification, weighting, and adjustment), regression that includes the PS as one of the adjusted variables has quantitative evidence as being the top performing PS method [47]. Finally, mixed effects regression further provides a powerful approach to causal inference. This mixed method contains both fixed effects (FE) and random effects (RE) components [48], with FE having the distinct advantage of controlling even for nonobserved traits by controlling for all time-invariant traits [41, 42]. FE does this by setting each individual subject as his/her own control and thus models within-person effects. Aside from the theoretical advantages, FE has shown quantitative strengths over competing methods [49–51]. RE was used to control for what FE cannot, namely, time-varying traits at the individual level with repeated observations over time (as students progressed through their medical education). The multilevel aspect was used to account for the hierarchical nature of the data in which medical trainees responded from different medical institutions. Inverse-variance weighted fixed effects (IVWFE) meta-analysis was used to produce a composite estimate across the 25 competency topics [52].

This triply robust approach can be represented by the following mathematical formula:

(1) $$\begin{array}{c} Pr\left(\;y_{ij}=1\;|\;u_{j}\;\right)=H\left(\;x_{ij}\beta +z_{ij}u_{j}\;\right) \end{array}$$

for *M* independent clusters conditioned on random effects *u*_*j*_ for *j* = 1,…, *M* clusters with cluster *j* constituting *i* = 1,…, *n*_*j*_ observations, outcome *y*_*ij*_, FE covariate *x*_*ij*_, RE covariate *z*_*ij*_, and regression coefficient *β*, all within a logistic regression equation with logistic cumulative distribution function of *H*(•) [53]. The PS is utilized as an additional variable in the above model and is represented as

(2) $$\begin{array}{c} e\left(\;x_{i}\;\right)=Pr\left(\;z_{i}=1\;|\;x_{i}\;\right) \end{array}$$

in which exposure probability is conditioned on the covariate *x*_*i*_ observed [54].

The competing causal inference methods of instrumental variable and difference in difference analyses were not utilized due to their methodological weaknesses, respectively, of having varying degrees of reliable instrumental variables and having a prevalence of unobserved confounders exerting time-varying effects before and after the exposure/treatment [55]. The above methods of doubly robust PS adjustment in multivariable regression were therefore integrated with multilevel mixed effects to provide a novel triply robust approach to causal inference. Aside from controlling for the likelihood of receiving GCCM education (via the PS) and unobserved time-invariant traits (via FE) and time variant along with intracluster correlation (via RE), this integrative method controlled via regression for age, gender, race, prior nutrition education, special diet, school year, intended specialty, and medical school. This multilevel approach has a Bernoulli conditional distribution of the response given the random effects and logistic cumulative distribution function for the success probability.

All results are reported as fully adjusted ORs. ORs rather than relative risks were calculated due to the complex data source described above and due to the rare disease assumption [56, 57]. Statistical significance was set at a two-tailed *p* value < 0.05. ML analysis was performed in R 3.3.2 (The R Foundation for Statistical Computing, Vienna, Austria). Statistical analysis was performed in STATA 14.2 (STATACorp, College Station, Texas, United States of America). Ethics approval was obtained through the Institutional Review Board (IRB) of Tulane University.

## 3. Results

Across the 20 medical education institutions nationally, 3,248 unique medical trainees met study criteria and produced 4,026 completed surveys over the 5 years of the study period. The mean (standard deviation, SD) age was 25.71 years (SD 2.91), 548 (61.43%) were female, 202 (22.65%) had nutrition education prior to medical school, 207 (23.21%) adhered to a special diet, 270 (30.27%) were in their clinical years, and 225 (25.22%) intended to enter a primary care specialty.

ML logistic regression for the primary endpoint indicated that GCCM versus traditional curriculum significantly improved student mastery of 25 competency topics (OR 1.97, accuracy 89.39%, RRSE 97.33%, and RMSE 0.30). Model performance was mildly boosted by using the top performing algorithm across the 43 tested, namely, simple logistic (OR 3.23, accuracy 89.32%, RRSE 97.10%, and RMSE 0.30).

Statistical analysis produced similar results with IVWFE meta-analysis of the estimates produced by triply robust propensity score adjusted multilevel mixed effects multivariable regression panel analysis. GCCM versus traditional curriculum significantly improved student mastery (OR 2.14, 95% CI 2.00–2.28, *p* < 0.001, accuracy 89.39%, and RMSE = 0.30) (Figure 1, Table 1). Notably, GCCM significantly improved every competency topic individually as well, with the largest improvements being in educating patients on the MedDiet (OR 9.41, 95% CI 4.73–18.75, and *p* < 0.001), low fat diet (OR 3.13, 95% CI 1.98–4.96, and *p* < 0.001), and fiber (OR 3.06, 95% CI 2.23–4.19, and *p* < 0.001), but also in nonnutrition areas including exercise (OR 1.72, 95% CI 1.34–2.21, and *p* < 0.001). In the first year of GCCM versus control exposure (with only Tulane University included as a study site), there was no significant improvement. By the second year with the same study site participating in CHOP, GCCM versus control significantly improved overall competencies (OR 2.52, 95% CI 2.06–3.08, and *p* < 0.001). Significance was retained with the scale-up of the intervention to 10 sites by Spring 2016 (OR 1.69, 95% CI 1.34–2.13, and *p* < 0.001), with increased magnitude of the association by Spring 2017 when 20 total sites were included (OR 2.14, *p* < 0.001).

GCCM versus traditional curriculum also significantly improved MedDiet high or medium versus low adherence (OR 1.40, 95% CI 1.07–1.84, and *p* = 0.015) and strong belief that nutrition counseling should be routine clinical practice (OR 2.56, 95% CI 1.95–3.38, and *p* < 0.001). GCCM also reduced by nearly half the odds of daily soft drink consumption (OR 0.56, 95% CI 0.37–0.85, and *p* = 0.007).

## 4. Discussion

CHOP-Medical Students is the largest known machine learning (ML) and causal inference statistical-driven multisite cohort study of nutrition education intervention for medical students. It is also the first known to utilize an evidence-based hands-on cooking modality to train future physicians in how to counsel patients on nutrition while improving their own diets. This trial provides robust evidence of causal inference across 3,248 unique medical trainees from 20 medical schools throughout America over 5 years that Goldring Center for Culinary Medicine (GCCM) hands-on cooking and nutrition education compared to traditional medical school curriculum improves student competency educating patients on 25 nutrition topics, in addition to students' own diet through increased Mediterranean Diet (MedDiet) adherence. The analysis features an innovative integration of artificial intelligence-based ML with advanced causal inference-based statistical methodologies that combines propensity score analysis with multilevel mixed effects regression within a longitudinal panel analysis, with both methods producing similar results. Additionally, the analysis suggests that this intervention is scalable and sustainable. Once the initial GCCM pilot program was optimized based on the latest research and student feedback after the first year, these significant improvements were first shown and then consistently shown despite scale-up of the program to 20 medical schools nationally with different cultures, resources, and infrastructures.

There are several important implications of these findings. First, these findings strongly suggest that the active teaching modality of hands-on cooking and nutrition education can significantly improve students' readiness to improve patients' health outcomes particularly for cardiovascular disease (CVD) through low-cost nutrition education. Second, these findings suggest that this intervention may add direct and indirect benefit to health systems, first by immediately improving patient outcomes through cooking classes taught by medical students and in the longer term by strengthening public health and medical sectors' capacities to bridge population health management and precision medicine through future physicians equipped with culinary medicine competencies. Third, these results provide evidence improving not only the quality of care delivered to patients, but also the societal equity of health outcomes. Not every patient can afford coronary stents, for instance, but the vast majority can afford optimizing their diet through practical tips physician can support them in implementing.

Next, this study suggests that this intervention is scalable and sustainable operationally, but also analytically. These results suggest medical students can be trained through GCCM classes to subsequently counsel patients in hands-on cooking and nutrition classes led by the same students who previously went through those classes as a supplement to the students' curriculum. Students and patients can benefit as the cost of such patient cooking classes can be kept low by students teaching the courses, financially backed by local health systems who could potentially benefit from having reduced costly hospital readmissions from the patients who would instead be better managed through their own optimized diets after the classes. Thus, by having results from well-established causal inference statistical analyses confirmed by the faster, automated ML algorithms, population health management and precision medicine can more accurately complement each other. Such ML algorithms can identify patient families most likely to benefit from the GCCM medical student-led cooking and nutrition classes, those classes can be provided to them, and the patients' health outcomes are tracked as they diffuse through their social networks as increasingly robust and diverse data sources including epigenetic and genomic data can be integrated into further refining the predictive accuracy and utility of those ML algorithms.

GCCM's larger CHOP cohort study is investigating the above implications currently through several Bayesian adaptive randomized trials. Phase I of the pilot randomized trial, CHOP-Diabetes, has previously shown that GCCM versus standard of care for patients with type 2 diabetes can significantly improve their diastolic blood pressure and total cholesterol, in addition to HbA1c (albeit nonstatistically significant for HbA1c in the smaller sample size of Phase I design) [58]. This trial suggested that lower-income residents from food desert communities had even greater benefit from the intervention than nonfood desert residents. Phase II trial of this study, CHOP-Family, has concluded study recruitment with forthcoming published results analyzing GCCM versus standard of care for parent-child pairs, while including a hospital readmission and cost effectiveness subanalysis. This analysis notably uses grocery store receipts not simply self-reported MedDiet consumption for improved study internal validity. If the target can be reached to improve family's MedDiet adherence by 30%, as the above CHOP-Medical Student analysis indicated GCCM can improve student adherence odds by 40%, then this could have substantive clinical and financial implications. Estruch et al. in the PREDIMED trial have prior shown in* The New England Journal of Medicine *that MedDiet adherence can reduce the likelihood of myocardial infarction, stroke, and cardiovascular disease- (CVD-) related mortality by 30% [15]. As CHOP-Family scales up to the other sites of CHOP-Medical Students, the question remains to be answered about how much of these health outcomes can be achieved and sustained in the long term through the classes.

These CHOP substudies are part of the larger CHOP cohort with a target recruitment goal of 19,500 subjects in its four tracks working toward establishing the evidence-based standard in nutrition education for medical professional, trainees, and patients: CHOP-Medical Professionals, CHOP-Community, CHOP-Employee, and CHOP-RCT (including Diabetes and Family substudies). This study features the GCCM moderated open curriculum, improved annually through the GCCM Annual Summit gathering physicians, dietitians, chefs, public health researchers, medical school administrators, industry representatives, and trainees from 45+ collaborating sites. This elective curriculum spans medical school, residency, physician continuing medical education (CME) credits, and community and patient cooking classes. Amid this collaboration since the center's founding in 2012 up to July 2017, GCCM has provided 53,674+ teaching hours to 4,051+ medical trainees/professionals and patients, including over 24,680 education hours to 444 medical students and 3,728 patients nationally.

## 5. Conclusion

CVD continues to be the most common and costliest mortality cause with the disease affecting nearly 1 in 2 patients, running up a bill of $555 billion annually [3]. Despite the well-accepted role of nutrition and other lifestyle interventions to reduce the incidence and disease impact on patients globally, physicians are ill equipped to respond to this growing global epidemic. As the incidence only is projected to increase with at least double the cost to $1.1 trillion over the next 20 years, American medical schools are faced with the growing need to train the next generation of physicians in evidence-based, equitable, scalable, clinical, and cost-effective interventions. This study, CHOP-Medical Students, provides the first known robust evidence of superior performance of a scalable nutrition education intervention for trainees, using a large, multisite prospective cohort, along with a novel integration of automated ML with causal inference statistics. Future studies are required to validate these findings internationally, in addition to concluding the ongoing nested Bayesian adaptive randomized trials within the larger CHOP cohort to determine clinical efficacy and cost effectiveness for patients when medical students teach their hands-on cooking and nutrition education classes. Such an interdisciplinary approach may accelerate the growing collaboration between the public health and medical sectors to bridge population health management and precision medicine to deliver the efficacious and equitable outcomes our patients deserve. The father of medicine, Hippocrates (460-370 B.C.), once asserted “let food be thy medicine and medicine be thy food” [59]. This study suggests that stretching into the future using our generation's latest artificial intelligence-based analytics and statistical methodologies and lifestyle interventions, we may together be discovering the wisdom of the past in making medicine what our patients deserve for it to be.

## Figures and Tables

**Table 1:** CHOP-Medical Students (*N* = 3,248): propensity score-adjusted multilevel mixed effects panel analysis showing medical students' improved competencies and diet.

Outcome		OR	95% CI	*p* value
Adherence	High/medium versus low			
	*Fall 2012–Spring 2016*	1.32	1.00*–*1.73	0.048
	*Fall 2012–Spring 2017*	1.40	1.07*–*1.84	0.015
	Olive oil	1.11	0.68*–*1.81	0.671
	Fruit	1.22	0.91*–*1.63	0.189
	Vegetables	1.13	0.84*–*1.52	0.416
	Vegetables/fruits	1.28	0.95*–*1.72	0.104
	Legumes	1.63	1.11*–*2.40	0.013
	Seafood	1.43	1.10*–*1.87	0.008
	Alcohol	1.09	0.71*–*1.67	0.694
	Meat	1.75	1.32*–*2.33	<0.001
	Whole grains	1.41	1.12*–*1.78	0.004

Daily intake	Fruit	1.22	0.91*–*1.63	0.189
	Vegetables	1.13	0.84*–*1.52	0.416
	Soft drinks	0.56	0.37*–*0.85	0.007

Strong agreement	Nutrition counseling should be routine	2.56	1.95*–*3.38	<0.001
	Specific counseling can improve patients' diets	1.73	1.38*–*2.17	<0.001
	Physicians counseling can improve patient's diets	1.62	1.31*–*2.01	<0.001

IVWFE meta-analysis	Total mastery of all 25 competency topics			
	*Fall 2012–Spring 2013*	0.98	0.81*–*1.19	0.859
	*Fall 2012–Spring 2014*	2.52	2.06*–*3.08	<0.001
	*Fall 2012–Spring 2016*	1.69	1.34*–*2.13	<0.001
	*Fall 2012–Spring 2017*	2.14	2.00*–*2.28	<0.001

Total competency counseling on	MedDiet	9.41	4.73*–*18.75	<0.001
	Dash diet	5.41	2.80*–*10.45	<0.001
	Vegetarian diet	3.04	2.05*–*4.52	<0.001
	Low fat diet	3.13	1.98*–*4.96	<0.001
	High protein diet	2.08	1.34*–*3.23	<0.001
	Serving size	2.79	1.93*–*4.04	<0.001
	Moderate alcohol	1.91	1.45*–*2.50	<0.001
	Eating disorders	1.80	1.29*–*2.51	<0.001
	Cholesterol	1.67	1.23*–*2.26	0.001
	Diabetes diet	2.52	1.85*–*3.43	<0.001
	Diabetes weight loss	2.05	1.56*–*2.68	<0.001
	Obesity weight loss	2.29	1.73*–*3.01	<0.001
	Omega fats	2.61	1.84*–*3.71	<0.001
	Dietary fats	2.72	1.70*–*4.36	<0.001
	Antioxidants	2.50	1.75*–*3.57	<0.001
	Calories	2.70	1.97*–*3.70	<0.001
	Hydration	1.73	1.34*–*2.24	<0.001
	Celiac	2.39	1.52*–*3.74	<0.001
	Food allergies	2.18	1.56*–*3.03	<0.001
	Glycemic index	2.29	1.54*–*3.40	<0.001
	Fiber	3.06	2.23*–*4.19	<0.001
	Food label	1.89	1.47*–*2.43	<0.001
	Osteoporosis	1.50	1.11*–*2.02	0.008
	BMI	1.46	1.13*–*1.88	0.004
	Exercise	1.72	1.34*–*2.21	<0.001

CHOP, Cooking for Health Optimization for Patients; GCCM, Goldring Center for Culinary Medicine; OR, odds ratio (logistic regression); 95% CI, 95% interval; IVWFE, inverse-variance weighted fixed effects; MedDiet, Mediterranean Diet; BMI, body mass index.
